# Ultra-high-resolution 3D digitalized imaging of the cerebral angioarchitecture in rats using synchrotron radiation

**DOI:** 10.1038/srep14982

**Published:** 2015-10-07

**Authors:** Meng-Qi Zhang, Luo Zhou, Qian-Fang Deng, Yuan-Yuan Xie, Ti-Qiao Xiao, Yu-Ze Cao, Ji-Wen Zhang, Xu-Meng Chen, Xian-Zhen Yin, Bo Xiao

**Affiliations:** 1 Department of Neurology, Xiangya Hospital, Central South University, Changsha 410008, China; 2 Department of Neurology, Xiangdong Hospital, Hunan Normal University, Zhuzhou 412200, China; 3 Shanghai Synchrotron Radiation Facility, Shanghai Institute of Applied Physics, Chinese Academy of Sciences, Shanghai 201800, China; 4 Center for Drug Delivery System, Shanghai Institute of Materia Medica, Chinese Academy of Sciences, Shanghai 201203, China; 5 Department of Pharmacy, Hunan University of Chinese Traditional Medicine, Changsha 410007, Hunan Province China

## Abstract

The angioarchitecture is a fundamental aspect of brain development and physiology. However, available imaging tools are unsuited for non-destructive cerebral mapping of the functionally important three-dimensional (3D) vascular microstructures. To address this issue, we developed an ultra-high resolution 3D digitalized angioarchitectural map for rat brain, based on synchrotron radiation phase contrast imaging (SR-PCI) with pixel size of 5.92 μm. This approach provides a systematic and detailed view of the cerebrovascular anatomy at the micrometer level without any need for contrast agents. From qualitative and quantitative perspectives, the present 3D data provide a considerable insight into the spatial vascular network for whole rodent brain, particularly for functionally important regions of interest, such as the hippocampus, pre-frontal cerebral cortex and the corpus striatum. We extended these results to synchrotron-based virtual micro-endoscopy, thus revealing the trajectory of targeted vessels in 3D. The SR-PCI method for systematic visualization of cerebral microvasculature holds considerable promise for wider application in life sciences, including 3D micro-imaging in experimental models of neurodevelopmental and vascular disorders.

Brain angioarchitecture and regional segregation of functional brain regions are key elements for advancing understanding on neurovascular functions in clinical research fields. High resolution imaging of 3-dimensional (3D) arrangements and structural parameters of the cerebrovascular network is undoubtedly an important prerequisite for the direct tracking of diverse neurobiological models in which cerebral perfusion is an issue. There has been great emphasis in recent years on establishing a 3D brain maps with outstanding global representation of vasculature at the ultra-high-resolution scale^1^,^2^. In particular, micro-architectonic probabilistic maps should enable the 3D identification of blood supply throughout the brain. Hitherto available rat brain angiography maps do not allow for the visualization and quantification of 3D angioarchitecture at high resolution. In fact, available imaging methods have been inadequate at the micron and sub-micron scale, principally focusing on the external 3D schema of vascular network instead^3^,^4^. In this context, in-depth anatomical resolution is an important pre-requisite to aid understanding of the cerebrovascular biology on a global-local scale, as well as to bridge the gap between 3D external imaging and endoscopic tracking of the cerebrovascular network.

To date, optical imaging techniques such as *ex vivo* two-photon laser scanning microscopy, or ultrastructural methods such as scanning electron microscopy (SEM) and transmission electron microscopy (TEM) have been available for visualizing tissues in 3D at the micrometer resolution range. However, these procedures are incapable of providing a brain-wide angioarchitecture map, and are ill-suited for providing 3D quantitative characterization at the microscopic level. Whereas two-photon laser scanning has limitations due to low penetration depth (<1 mm)^5^,^6^, electron microscopic methods are incompatible with studies in the living organism or intact brain. The recently developed micro-computed tomography (CT) technique serves to differentiate the 3D connectivity of anatomical vasculature within intact brains in rodents. However, this method is constrained by its inherently limited resolution to detect only those micro-vessels measuring approximately one micron in diameter^7^. In particular, the imaging methods based on vascular disease are reliant upon distribution of injected contrast agents throughout the vascular endothelium. Incomplete filling and leakage of contrast media will inevitably detrimentally affect the quality of the images obtained. However, the recent advent of state-of-the-art synchrotron radiation (SR) facilities has usurped the diffraction limitation of conventional optical imaging techniques, by providing brilliant and partially coherent radiation suitable for ultra-high-resolution imaging of thick samples^8^. Remarkably, SR-based phase contrast imaging (SR-PCI) has already proven especially appropriate for 3D visualization of high image contrast fine microstructures in biological tissues that have low X-ray absorption without the addition of contrast agents^9^. A recent report^10^ has demonstrated that SR-PCI allows the simultaneous visualization of 3D micro-vascular network and neuronal systems in *ex-vivo* mouse spinal cord at sub-micron resolution, and without requirement for any contrast agent. Hence, SR-PCI seems poised to emerge as a powerful tool in depicting the 3D arrangements of cerebral vasculature, in conjunction with a global-local perspective supported by the phase shift information generated by vessels within the volume.

In order to satisfy the aforementioned requirements, we have set out to define ultra-high-resolution 3D digitalized angioarchitectural maps of rodent brain. The perspective of the brain surface volume and intra-cerebral visualization are presented, taking advantage of the computational methods required for analysis of large SR-PCI image data sets. Typical image volumes can comprise for each sample 12 GB, including original projection and slice data. In particular, we reconstructed multi-angle 3D digital anatomical images revealing the spatial network of vessels in specific brain regions including the hippocampus, pre-frontal lobe cortex and corpus striatum. Furthermore, we attempted to perform synchrotron-based virtual micro-endoscopy of cerebral vessels, so as to dynamically track the pathway of targeted vessels from the luminal side. Additionally, we acquired substantial 3D quantitative information about vessel content by means of analysis of global network skeleton maps. To the best of our knowledge, the present work takes the lead in systematically presenting 3D brain-wide angioarchitectural maps of high quality based on the SR-PCI technique, as well as providing a description of synchrotron-based virtual micro-endoscopy and dynamic visualization of navigation through the vasculature. This achievement paves the way for a wider application of systematic and in-depth investigations of 3D micro-angioarchitecture on a global-local scale in diverse biological states, and will aid pathophysiology models to be developed. The newly-established 3D image processing and analysis method for complicated vascular networks may act as a catalyst to inspire further non-destructive imaging studies on vascular micro-morphology using SR-PCI methods.

## Results

### Hierarchical image processing

**Figure 1:**
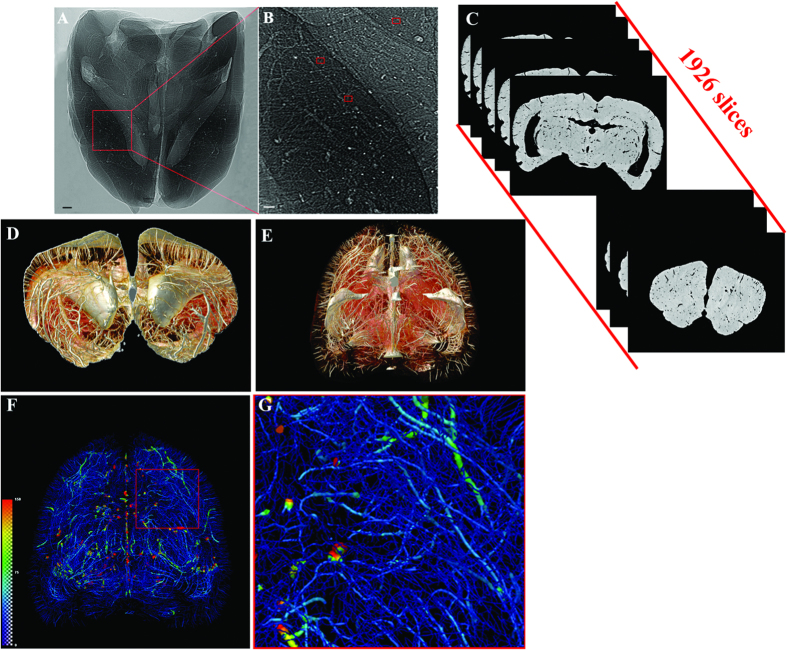
Illustration of hierarchical image processing. (**A**) Projection of rat brain by SR-PCI. (**B**) Local magnification of the region of interest denoted by a red box in (**A**). The small red frame in (**B**) indicates vessels with diameter of approximately 10 μm. (**C**) Series of 2D reconstructed slices using projections with image optimization. (**D**) 3D local tomography of angioarchitecture via superimposition of slices. (**E**) 3D reconstructed image of cerebral vasculature. (**F**) 3D skeleton of vascular network (in pseudocolour), with the region of interest denoted by a red frame. This area is shown with higher magnification in (**G**). The color gradients reflects vessel diameters, ranging from 10 μm (dark blue) to 150 μm (red). Scale bars: 200 μm (**A**,**B**).

We established a systematic and highly effective approach to the processing of large brain datasets derived from the original projections of optimized 2D slices to the ultimate 3D high resolution images. The initial 2D projections of ultra-high contrast were the important foundation for successive analysis. In Fig. 1A,B, the contours of the cerebral vasculature are clearly depicted via effects of edge enhancement. The smallest vessels that could be identified, as shown in the red frame, were approximately 10 μm in diameter. The optimized slices can provide microstructural information at different section levels (Fig. 1C). Subsequently, partial or integral slice-by-slice reconstruction can yield 3D images of the specific brain regions or of whole brain (Fig. 1D,E). The vascular network composite was then extracted and represented with pseudo-color for quantitative analysis (Fig. 1F). Enlargement of the image demonstrated that microvessels measuring <30 μm in the region of interest are arranged in a continuous and dense criss-cross pattern (Fig. 1G).

### 3D surface volume rendering images of brain

**Figure 2:**
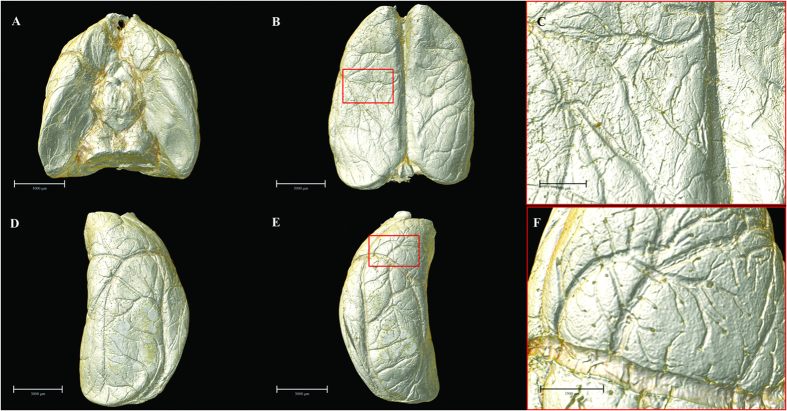
Multi-angle surface-rendering of the 3D reconstructed brain. (**A**) Ventral view. (**B**) Dorsal view (**C**) Partial enlargement view of (**B**) with red box showing the leptomeningeal anastomotic branches. (**D**) Lateral right view. (**E**) Lateral left view. (**F**) The pial branches of middle cerebral artery. Scale bars: 3000 μm (**A**,**B**,**D**,**E**), 1000 μm (**C**) and 1500 μm (**F**).

By means of 3D surface volume rendering, the present data authentically outlined vascular trees at the brain surface from multiple perspectives (Fig. 2). Without any use of contrast agent, SR-PCI can thus provide image detail sufficient to clearly show the blood supply at the cortical surface in 3D. The leptomeningeal rami anastomoticus and the multiple pial branches of the middle cerebral artery (MCA) were also well identified, which is propitious for future use of the SR-PCI in studying collateral circulation in diverse biological states.

### Ultrahigh-resolution 3D digitalized anigoarchitectural map

**Figure 3:**
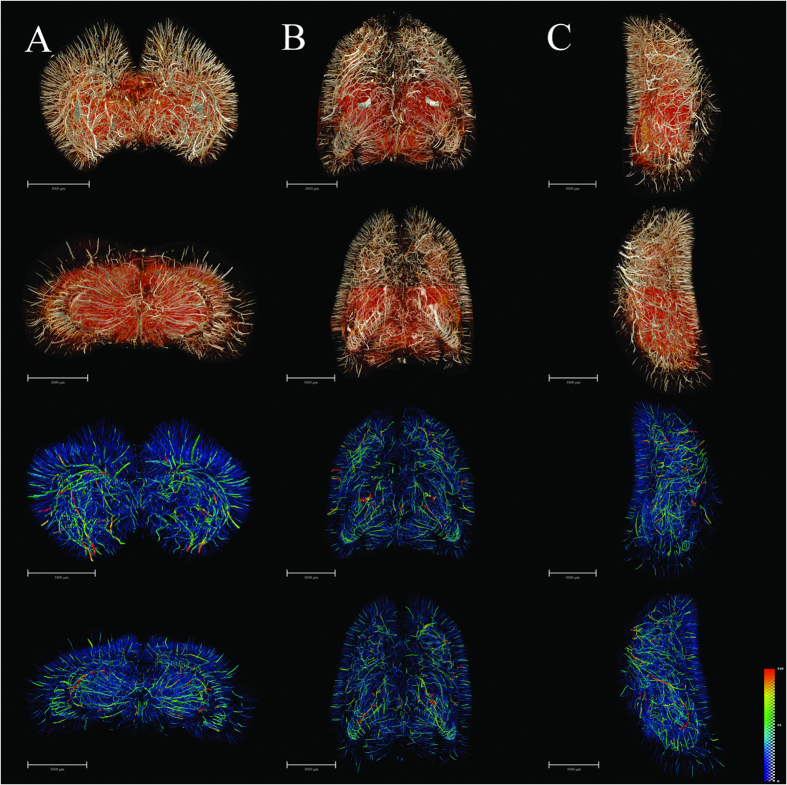
3D tomography of cerebral angioarchitecture in the three planes. (**A**) Rostral (top) and caudal (upper-middle) views of vascular mapping. 3D corresponding skeletonization in rostral (lower-middle) and caudal (bottom) views. Pseudo-colored enhanced effects represent the distributional range of the vessel diameter spectrum. (**B**) Dorsal (top) and ventral (upper-middle) views of vascular mapping. 3D corresponding skeletonization in dosal (lower-middle) and ventral (bottom) views. (**C**) Lateral right and lateral left views of vascular mapping. 3D corresponding skeletonization in lateral right (lower-middle) and lateral left (bottom) views. The pseudocolor bar in the lower-right corner of C panel indicates the diameter ranges of vascular trees in the whole brain. Scale bars: 3000 μm (**A**–**C**).

We obtained very detailed cerebral 3D digitalized angioarchitectural maps (Fig. 3). No dislocation was observed along the dorsal-ventral, rostral-caudal and right-left lateral axes in the reconstruction of the 3D cerebral images. In other words, the slices in the three planes were inherently aligned. In particular, we managed to process the images to remove cerebral ventricles, thus eliminating an important source of the visual interference, and giving a clearer depiction of the complete spatial vascular network. Herein, we see the penetration of cortical arteries vertically into the cortex and terminating in different cortical layers. Typical short, intermediate, long cortical, subcortical and medullary vessels were clearly distinguished. The vascular network tended to be more abundant in the deep cerebral cortex than in superficial layers. Vessels in the medulla mostly exhibited heliciform or corrugated arrangements, forming substantial obtuse or right-angled branches in the brain parenchyma. Additionally, corresponding 3D vascular network skeleton maps were extracted for subsequent analysis. Continuous pseudocolour alternations depicted the distribution range of the spectrum of vessel diameters, thus indicating that vessels with diameter <30 μm (depicted in blue) form a key component of brain vasculature.

**Figure 4:**
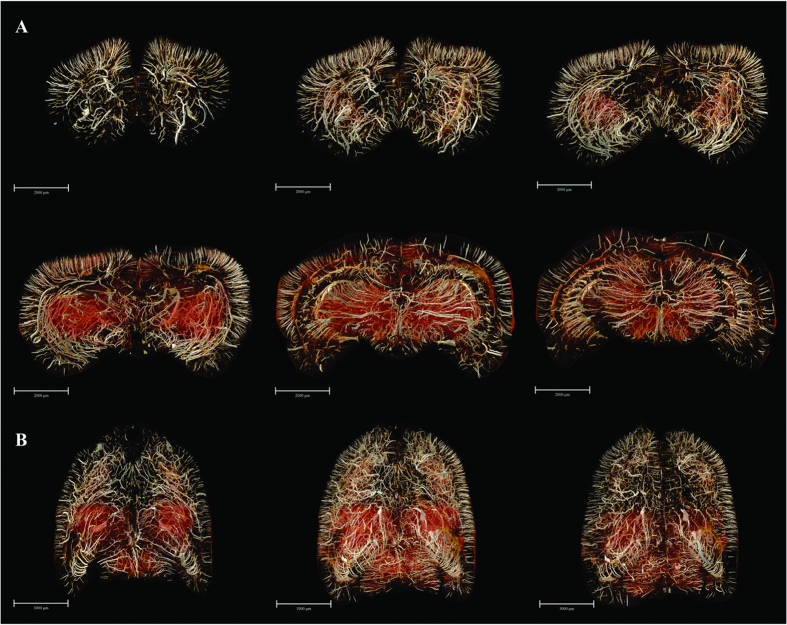
3D digitalized angioarchitectural map. (**A**) A series of mapping from frontal pole to the upper midbrain, which were separately reconstructed in 3D using a stack of 100 coronal slices (total thickness of 592 μm). The cerebral ventricles were removed to delicately describe the panorama of vascular architectonic features. (**B**) Overviews of the whole-brain maps in the 3D-reconstructed horizontal planes from 200 serial slice-by-slice rendering respectively. Scale bars: 2000 μm (**A**), 3000 μm (**B**).

**Figure 5:**
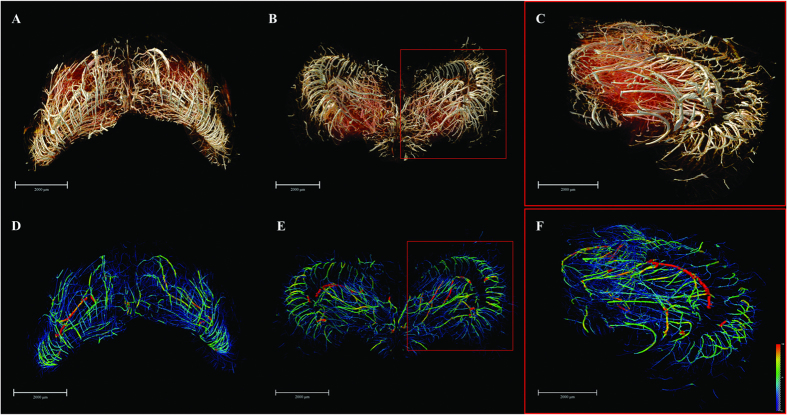
The 3D angio-architecture and skeletonization of the hippocampus. (**A**,**D**) Dorsal view. (**B**,**E**) Ventral view. (**C**,**F**) The higher magnification of the unilateral hippocampus is presented in the red box in (**B**,**E**). The pseudocolor bar in the lower-right corner of F panel indicates the diameter ranges of vascular trees in hippocampus. Scale bars: 2000 μm (**A**–**F**).

**Figure 6:**
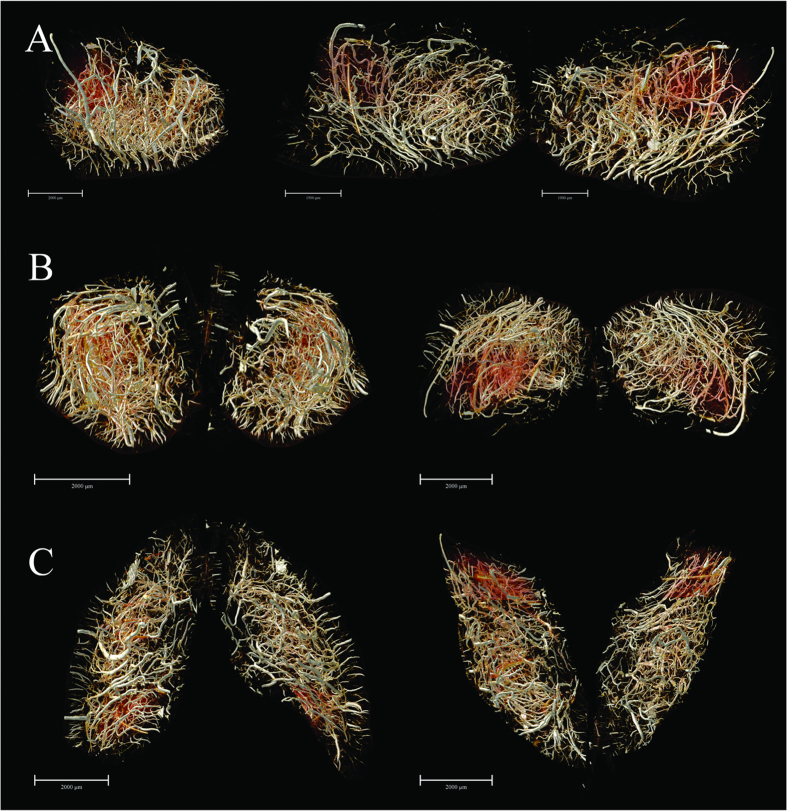
The 3D digitalized maps of vasculature mainly supplying the pre-frontal lobe cortex and corpus striatum region (defined as the region of interest, ROI) in the three spatial axes direction. (**A**) The lateral view. Overlapping of bilateral vascular trees form a complicated spatial micro-structure (left). The separate right side view of the ROI (middle). The separate left hand view of ROI (right). (**B**) 3D visualization in the rostral-caudal axis. Rostral view (left) and caudal view (right). (**C**) 3D visualization in the dorsal-ventral axis. Dorsal (left) and ventral (right) view. Scale bars: 2000 μm (**A** left **B**,**C**), 1500 μm (**A** middle, **A** right).

Subsequently, we acquired a series of vascular maps extending from the frontal pole to the upper midbrain, which were separately reconstructed in 3D using a stack of 100 coronal slices (to a total thickness of 592 μm) (Fig. 4). In addition, the overviews of the whole-brain maps in the 3D-reconstructed horizontal planes were rendered. Thus, we could observe the 3D micro-morphology predominating in the brain areas, which allowed for detailed analysis. An innovation arising from our study consists of the extraction of multi-angle views of angio-architectural networks of anatomically distinct functional brain regions, with emphasis placed on the hippocampus, pre-frontal lobe cortex and corpus striatum (Figs 5 and 6). Subtle details of the 3D distribution of the blood supply in these structures emerged from our analysis. Compared with corpus striatum, the vessel organization in hippocampus was arranged in a relatively regular pattern (Fig. 5). The larger vessels were mainly located around the peripheral area of the hippocampus, thus forming a shell-like 3D shape, while smaller vessels were distributed mainly within the inner domain. Significantly, vessels in the pre-frontal lobe cortex and corpus striatum were arranged in a more convoluted manner than in the hippocampus (Fig. 6).

### 3D quantitative characterization

**Figure 7:**
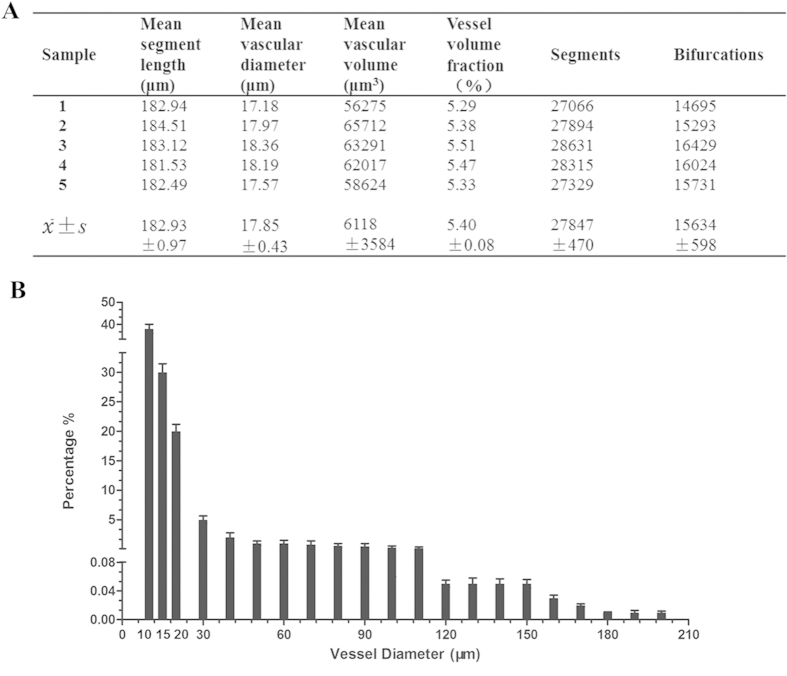
3D quantitative characterization. (**A**) Morphometric parameters based on global vascular network analysis. (**B**) The percentage distribution in quantity of global vessels at different diameter ranges.

The most important parameters that emerged as being closely related to the cerebral angioarchitectural profile are listed in Fig. 7A. The fractional frequency distribution of different blood vessel size ranges was also analyzed (Fig. 7B). These results demonstrated that the cerebral vessel branches were extraordinarily numerous, and the abundance of blood vessels significantly increased with decreasing vessel diameter. Approximately 90% of vessels were <30 μm in diameter, and had a distribution correlating highly with the overall structure of the microcirculation, as defined by the scaffold of large vessels.

### 3D virtual cerebrovascular micro-endoscopy

**Figure 8:**
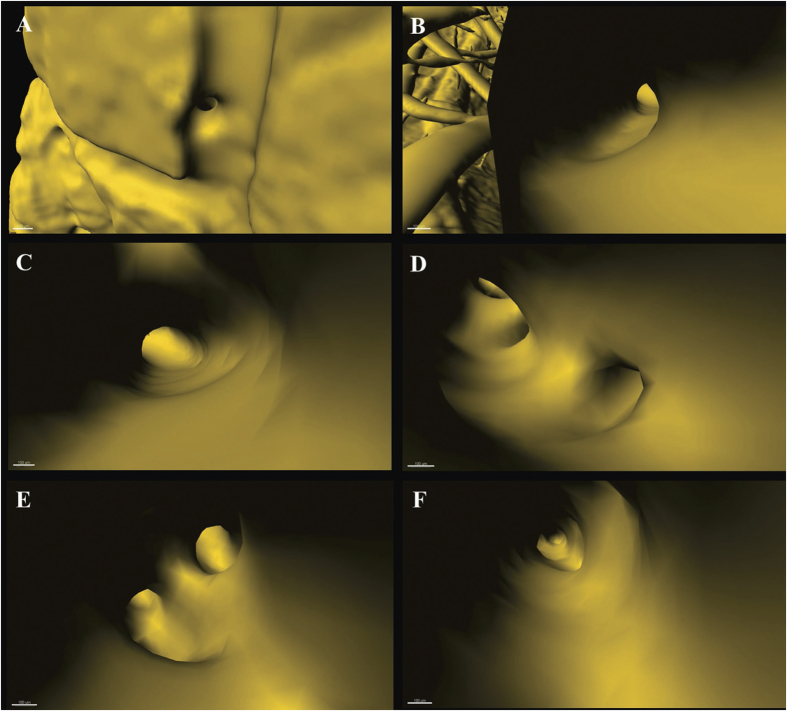
Virtual micro-endoscopy to 3D track targeted vessel. (**A**) Initiation to orientate a vessel. Successive pathway tracing shown in (**B**) to (**F**). The endovascular micro-structure is clearly discernible. Scale bars: 100 μm.

Virtual micro-endoscopy offers a novel perspective for observations within the intravascular space. The 3D micro-structural features of the targeted vascular lumen could be identified (Fig. 8). Concurrent application of 3D virtual flight through the brain space was possible with automatic navigation through the vessel lumen and constitutes a powerful tool for stereoscopic visualization and measurement of endovascular anatomy (Supplementary movie 1).

## Discussion

A proper mapping of the angioanatomical architecture is considered to be the pivotal foundation for advancing our understanding of cerebrovascular function and dysfunction. A pre-requisite to resolve the cerebral angioarchitecture at high resolution and accuracy is to develop a powerful imaging tool, which is sufficiently sensitive to penetrate optically opaque brain tissue for the detection of vessels at the micron scale. Considering the intricate and convoluted nature of the vascular network, conventional 2D histological sections are unsuited to provide sufficient data compared to 3D imaging in terms of morphology and morphometry^11^,^12^,^13^. Despite the high magnifications needed to visualize tissues for 3D studies, modern advanced optical imaging techniques are generally restricted to observing transparent or fluorescent objects, and this with a limited view field or low penetration depth (<1 mm)^14^. This undoubtedly imposes exacting requirements for sample preparation. Existing imaging approaches such as MRA, CTA and Micro-CT all depend on the application of a contrast medium. Consequently, any imperfect perfusion or delivery of contrast medium can perturb the results^7^,^15^. The state-of-the-art SR-based phase contrast imaging method enables one to steer clear of these issues, which is a particular advantage for soft biological tissues which require 3D imaging with high spatial resolution and short exposure time, and is also an issue relevant for high sample throughput^15^,^16^,^17^.

The present study exploited new SR-PCI techniques initially to establish a cerebral 3D microvascular connectivity map with ultra-high resolution, without destroying the sample in the process. In principle, 2D projections with greatly enhanced contrast provide the basis for obtaining superlative 3D images. For this PCI method, it is crucial that the vascular structural information produced by well-resolved spatial gradients of image brightness is maximized. By means of the “edge enhancement” effect^18^, vascular interfaces can be highlighted due to the presence of effectively amplified gradients induced by in-line phase contrast measured at appropriate propagation distances. Accordingly, the high-quality of data arises from the integrated optimization of distance, photon flux, high signal-to-noise ratio, and less blurring of the Fresnel diffraction patterns^14^,^19^. In our successive data analysis pipeline, we combine noise reduction filtering, phase retrieval algorithms, 3D reconstruction, 3D quantitative analysis, and 3D virtual micro-endoscopy tracking to extract phase contrast information of vessels at the utmost limit of technical capabilities.

In a comparable study by Heinzer *et al.*^13^,^20^, hierarchical microimaging with microcomputed tomography (μCT), synchrotron radiation μCT (SRμCT) and SEM were used to realize top-down visualization of brain vasculature; their approach was based on vascular corrosion casting (VCC) using a synthetic polymer to fill the vasculature, with elimination of the surrounding brain parenchyma. Our present experiment differs from the previous study in several important aspects. First, we demonstrated enhancement of vascular features using PCI without the need for contrast agents. Compared to present methods, the VCC preparation is quite complicated. In order to obtain high quality castings, demanding requirements for the type, quality and total volume of the contrast medium, and perfusion must all be met. In particular, excessively high injection pressure can easily damage brain capillaries, thus leading to local leakage of contrast agents, whereas lower pressure can cause incomplete and inhomogeneous filling of small vessels, especially brain capillaries. Any imperfection in these procedures will have deleterious effects on the accuracy of vascular imaging. In our relatively more simple and non-destructive preparation tissue process, the delicate structures of the microvessels are better preserved, while introducing high contrast air boundaries. Further to this, *ex vivo* brain sample preparation for current μCT or SRμCT scanning procedures generally includes successive steps of perfusion with paraformaldehyde, injection of contrast agents, fixation and finally dehydration. In practical terms, scanning requires that final samples be dried fully following the fixation step, while appreciating that this may potentially introduce some minor deformation of the sample. While this inevitably arises to some extent with 3D visualization of brain vasculature *ex vivo*, published papers show that the extent of this deformation is generally acceptable with existing methods^21^,^22^,^23^. As in-line phase-contrast imaging derives image contrast from refractive index gradients, it is ideally suited for using air as a contrast agent. We concede that blood-containing samples fixed in formalin, especially when prepared in phosphate buffered saline (PBS), but without additional contrast agents, should be a state closer to that reflecting normal physiological conditions. In theory, the original morphological features should thus be better retained than is the case with more invasive processing methods. Consequently, we are now testing alternate tissue fixation agents closer to conditions *in vivo*, with an aim of reducing further potential structural changes during the dehydration process. Second among the innovations and advantages of our approach, we note that a particular requirement of VCC is to dissolve the brain parenchyma with special chemical reagents, while retaining the vascular framework. In contrast, we retain the intact brain, which seems more amenable for observing precise anatomical relationships between vasculature and tissues. For example, in the ischemic stroke model, we are now testing PCI to identify the fine structural changes of the infarct region and its ischemic penumbra, and also follow post-stroke angiogenesis, without technical complications due to leakage of contrast agents, or conversely incomplete filling of the lumen. Moreover, Heinzer *et al.* obtained the whole brain image by μCT with resolution of 16 μm, while SRμCT yielded in our hands a final pixel size of 5.92 μm, Thus, 3D visualization of the whole brain angioarchitecture in the present study was evidently globally superior than in the earlier study. Notably, we extracted ROIs for hippocampus and corpus striatum, thus laying the foundation for further study of regionally distinct pathological vascular alternations, thus meeting the requirements for models of neurodegenerative disorders and ischemic stroke respectively. Finally, we note that use of contrast agents in some previous studies^24^,^25^ impedes subsequent, histological analysis, such as HE staining. Following PCI scanning, our samples can be re-used for follow-up histological studies, thus affording verification or comparison between different methods.

The present findings and data notably reveal the multi-stage pial vascular plexus, as well as the fine intra-cerebral microvascular structure with sub-10-μm spatial resolution. Even at more discriminatory levels, the complex 3D angioarchitectural maps at the global and different sections levels are displayed with great detail in multiple spatial perspectives. From the architectonic view, we describe the 3D features of different types of cortical and medullary vessels. In this regard, most branches of medullary vessels are seen to emerge at obtuse angles or right angles, and the spiral orientation we observe may lead to changes in hemorheology in the living organism, thus resulting in turbulence of local blood flow, which is potentially harmful to the vascular endothelial cells^26^,^27^. The present work thus provides potential imaging-based evidence for elucidating the precise nature and mechanism of microcirculation hemodynamics. Remarkably, the angioarchitectural mapping of particular brain regions i.e. the hippocampus, pre-frontal lobe cortex and corpus striatum were separately extracted and presented. With respect to architectonics, our work reveals for the first time the integrated microvascular connectivity of ROIs precisely via SR-based 3D visualization. The observed distinctions in 3D global vascular topography imply that the connectivity is not merely a purely geometric phenomenon, but that microvessels maintain the principal scaffold framework of the cerebral vasculature. It is well known that once the brain suffers from ischemic injury, microcirculation will respond in a compensatory manner^8^. We find that the combination between 3D topography and quantitative characterization can provide considerable qualitative and quantitative information. This systematic approach to analysis is expected to be applicable for comparison of vasculature parameters under various pathological conditions, aiming at revealing visible and measurable proof of altered microvasculature architecture in the targeted region. We utilized SR-based virtual micro-endoscopy to track in 3D, the pathway of a targeted vessel. This enables visualization of the micro-structure of the vascular lumen and inner walls, and even allows measurement of the internal diameter of selected vessels. This innovation will make it possible to localize the relevant blood vessels and non-invasively measure pathological changes in various models of vascular diseases, and should also provide a potential way to monitor and target drug delivery in functional vascular mapping studies.

All in all, the established systematic method should be readily applied to other studies in different pathological models, and this holds significant promise as an effective analytic technique for non-destructive evaluation of diverse pathological vascular alternations. As noted above, we are now using these methods to study angiogenesis after ischemic stroke; initial results are promising, imparting abundant information on vascular morphological features in the infarct region, ischemic penumbra and even distant sites. Furthermore, we are attempting to resolve the 3D morphometry parameters of ROIs, which should prove useful for evaluation of potential therapeutic strategies for stroke.

Although the SR-PCI technique has certain outstanding advantages, it does suffer from certain limitations. Thus, its large field of view (FOV), resulting in a large sample volume, can only be acquired with low magnification and resolution, and vice versa. For example, in order to reveal capillaries with diameter about 1.5 μm in images with a pixel size of 0.74 μm, the standard FOV arising for this resolution is around 3 mm, which is inadequate for the whole mouse brain. For relatively large specimens like rat brains, only small portions can be imaged in this manner. Since our present study principally concentrates on establishing 3D imaging and analytic methods of brainwide vasculature, whole brain sample imaging is obtained with a certain sacrifice in resolution. Thus, capillaries 4–7 μm in diameter fell under the detection threshold with our 5.92 μm resolution detector. In future studies, we will apply higher resolution detectors for observation of focal microvasculature. Another issue is that very few studies have hitherto addressed the 3D identification of some intrinsic parameter to quantify vessel density globally in the whole brain. Available studies with 3D analysis have rather investigated vascular density parameters only in some small ROI, giving the number of vascular profiles per unit area. SR-PCI data is demanding for storage, a dataset for each sample consists of 12 GB, including original projection and slice data, which requires 512 GB RAM for practical reconstruction and measurement. Additionally, it requires sophisticated data processing for visualization and especially for quantification. It is not yet available for a software-based automated quantification of vasculature parameters in the SR-PCI datasets from entire brains. In the present study, we attempt merely to develop a systematic 3D analysis and quantification method for the parameters mentioned, in preparation for still more ambitious studies. Some of the vessel parameters (Table A) are not completely in agreement with the results from a previous SR-PCI study. To our knowledge, Heinzer *et al.*^20^ performed 3D quantification of microvasculature in cubical brain samples measuring 1 mm^3^ cube using an SR technique at a resolution of only 1.4 μm per pixel. Their estimate of mean vessel diameters in wild-type mice of 3.4 μm is in close agreement with our results, although their vessel volume fraction was slightly smaller. We consider the resolution effects and also the rodent strains may account for these differing results. Furthermore, our study calculated the global vasculature parameters in the entire brain, whereas their results are for particular brain regions examined with a small ROI to calculate vascular profiles per unit area at a higher resolution.

In summary, our study established a systematic analysis strategy for cerebral mapping of angioarchitecture based on the SR-PCI technique, which provides a basis for quantifying anatomical topology of the vascular network in rat brain at the micrometer scale. The presented method should be applicable to a broad range of life science applications concerning vasculature, and should emerge as an important resource for generating accurate and precise input parameters for modeling and simulation.

## Methods

### Animals

Animal care and use was performed in accordance with the guidelines of the Administration Committee of Affairs Concerning Experimental Animals in Hunan Province, China. All experimental protocols were approved by the Animal Ethics Committee of Central South University, Changsha, China. A total of five Sprague–Dawley® (SD) male rats (220–250 g) were obtained from the Animal Center of Central South University, Changsha, China. Animals were housed in a temperature-controlled room with a 12-h light/dark cycle with free access to food and water and acclimatized for at least seven days before use.

### Sample preparation

Rats were deeply anesthetized using an intraperitoneal (i.p.) injection of 10% chloral hydrate (0.4 ml kg^−1^) followed by desanguination of the brain. Subsequently, phosphate buffered (pH 7.4) 4% formalin was perfused trans-cardially for tissue fixation. All the perfused solutions were preheated to 37 °C, and the procedure was conducted in a laboratory at 22 °C. Rat brains were then extracted and post-fixed at 4 °C for 24 hours with 4% paraformaldehyde in PBS. The rinsed brain specimens were then dehydrated by sequential immersion in 50%, 70%, 85%, 95%, 100% alcohol, each for 24 h, and were finally were dried in air. Thus, brains were in a fully dehydrated state for SR-PCI.

### SR-PCI data acquisition

Images were captured at the BL13W1 beamline of the Shanghai Synchrotron Radiation Facility (SSRF, China). X-rays were generated from an electron storage ring with an accelerated energy of 3.5 GeV and an average beam current of 200 mA. The beamline covered a tunable energy spectrum ranging from 8 to 72.5 keV. After being monochromatized with a double-crystal monochromator equipped with Si(111) and Si(311) crystals, the transmitted X-rays were captured by a 100-μm thick cleaved single-crystal CdWO4 scintillator, and converted to a visible image. Samples were placed on a target 34 m downstream to the SR source, and the distance between the sample stage and the detector ranged from 0 to 8 m.

After dehydration, the brain had a width of about 9 mm and height of nearly 10 mm. In consideration of the appropriate size of the acquisition window, the ×1.25 magnified lens was selected, which has a maximum acquisition width of 11,443.36 μm (1933 pixels); this is sufficient to cover the sample in the horizontal axis. The corresponding height of the capturing area was 5,700.96 μm (963 pixels), and the sample was consequently scanned twice at the upper and lower half, with an overlap of about 200 pixels. The immobilized samples were placed at the center of the rotation stage, and scanned with synchrotron radiation X-rays at 20.0 keV. After penetrating the sample, the projections were magnified using diffraction-limited microscope optics (×1.25 magnification), and digitized using a high resolution 2,048 × 2,048 pixel CCD camera (pco.2000, PCO AG, Kelheim, Germany). The voxel size was 5.92 × 5.92 × 5.92 μm^3^, the exposure time was 2.2 s and the sample-to-detector distance was 50 cm. For each acquisition, 720 projections over 180° were collected. Light field images (i.e. X-ray illumination on the beam path without the sample) and dark field images (i.e. X-ray illumination off) were also recorded during each acquisition, to eliminate possible electronic noise and variations in the brightness of the X-ray source.

### 2D Slice reconstruction and optimization

The total projected images for the samples were reconstructed using the software X-TRACT SSRF CWSx64, which was developed by CSRIO (Commonwealth Scientific and Industrial Research Organization, Australia, http://www.ts-imaging.net/Default.aspx), and performs a direct filtered- back-projection algorithm. To enhance the quality of reconstructed slices, propagation-based phase contrast extraction was carried out. The X-TRACT SSRF CWSx64, which performed the TIE-HOM algorithm, was used for the recovery of the optical phase of an electromagnetic wave from a single near-field in-line image by solving the Transport of Intensity Equation (TIE). For the principle of phase contrast imaging, the amplitude and phase of X rays were affected by the interaction of the wave with matter, and the forward diffraction can formally be described by the complex refractive index n of the medium as


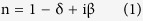


The refractive index decrement δ results in a phase shift, and the absorption index β is linked to the linear absorption coefficient. During phase extraction, the parameter of δ/β was adjusted to 400 according to preliminary experiment. After phase retrieval and reconstruction, intensity scales were truncated, with rescaling to a grey value ranging between 0 and 255. A total of 1,926 reformatted slices were obtained for each sample; the resolved images had a high quality phase contrast. Based on the 2D slices, micro vessels in the scanned sample could be distinguished within the parenchyma. Two radiographs at the same angle from the upper and lower half of the sample were selected, and the background was subtracted for noise correction. Then a tiling algorithm based on Fast Fourier Transformation translation correlation was performed to calculate the exact overlapping area of the two hemi-brain CT scans. After the overlapped slices were removed, the slices from the two half samples were merged and aligned to correct for offset angles.

Subsequently we performed grey level-based segmentation with an algorithm implemented in an in-house program, thus discriminating voxels within vessels and voxels within parenchyma. Moreover, phase contrast information of vessels was significantly emphasized and subsequently transformed into binary mapping for further 3D analysis.

### 3D visualization

The 3D rendered images were first reconstructed as a sequential series of 2D slices, and then the 3D rendered data were analyzed with the commercially available software VG Studio Max (Version 2.1, Volume Graphics GmbH, Germany), Image Pro Analyzer 3D (Version 7.0, Media Cybernetics, Inc., USA), and Amira (Mercury, Richmond, TX, USA). We developed both in-house manual and automatic registration to reveal multi-angular 3D angioarchitecture data, to obtain qualitative and quantitative results respectively.

After completion of the above steps, the regions of interest (ROIs) within the 3D model from the rendered sample were re-sliced to determine their structural morphology. The parenchymal volume in the sample was extracted using grey value-based segmentation, with an in-house-programmed algorithm. In contrast, microvessels in the sample were shown in this format as the inner void, and have the same absorption coefficient as the space outside the sample. In order to extract the morphology of the vascular network, the segmented images were inverted to highlight vessels. A morphological filter algorithm was then performed to distinguish the micro-structure of vessels within the void space in the sample. Finally, 3D models of both the vessels and the parenchyma were rendered and colored with different colormaps; the autumn color map for indicating brain parenchyma varies smoothly from red, through orange, and to yellow. The jet color map used to depict vasculature ranges from blue to red, and passes through the colors cyan, yellow, and orange, to indicate increasing density.

### Network analysis

The 3D networks of vessels extracted from rendered data were visualized and characterized with a dendrite analysis method based on morphological algorithms, which include parameters for thinning, distance mapping, skeleton and vectorization. To quantitatively resolve the vascular network, we undertook the following protocol: (1) skeletonization, that is to say thinning of the binary input vasculature for centerline extraction, which contains directly interpretable information, and provides a distance map; (2) analyzing the skeletonized image. Here, the branches and branch-points were determined and vessel parameters were calculated, including bifurcations, segments, length, vessel volume, vessel volume fraction and distribution of vessel diameter. A segment was defined as any section of vasculature connecting two vessel bifurcations, between a bifurcation and a vessel end point, or between two non-connected segment-end points. In this analysis, the vessel end points were excluded from the sum of bifurcations. Vessel volume fraction referred to the ratio between the number of voxels belonging to vasculature and the total number of voxels in the sample. A statistical analysis was also carried out. The final part of the protocol included global morphometry. By means of extraction of the vascular network skeleton, quantification of parameters such as length, diameter, volume for each segment, and global statistics were carried out. All of the 3D quantitative analysis was performed with the commercially available Image Pro Analyser 3D software (Version 7.0, Media Cybernetics, Inc., USA). The data are reported as the mean ± standard error.

### Virtual cerebrovascular micro-endoscopy

By virtue of 3D reconstruction, we further attempted to perform virtual cerebrovascular micro-endoscopy based on surface rendering. The process included path planning and real-time rendering. The key issue was to simulate virtual camera recordings, while moving dynamically through a targeted vessel lumen. After the 3D rendered ISO-surface model had been generated and exported as a Virtual Reality Modeling Language (VRML) model, the surface model was visualized in the 3D constructor module of Image Pro Analyzer 3D. With the Animation feature, the Animation path from the list of 3D elements, which includes items such as circular animation, cutting and slicing, were selected to create and record the animation files. Setting the position and orientation of the camera and changing it along the pathway within the vessel, “virtual flight” was realized for the visualization of vessels from this novel perspective.

## Additional Information

**How to cite this article**: Zhang, M.-Q. *et al.* Ultra-high-resolution 3D digitalized imaging of the cerebral angioarchitecture in rats using synchrotron radiation. *Sci. Rep.* **5**, 14982; doi: 10.1038/srep14982 (2015).

## Supplementary Material

Supplementary Video Legend

Supplementary Video 1

## References

[b1] Li A. *et al.* Micro-optical sectioning tomography to obtain a high-resolution atlas of the mouse brain. Science 330, 1404–1408 (2010). 21051596 10.1126/science.1191776

[b2] Amunts K. *et al.* BigBrain: an ultrahigh-resolution 3D human brain model. Science 340, 1472–1475 (2013). 10.1126/science.1235381 23788795

[b3] Pathak A. P., Kim E., Zhang J. & Jones M. V. Three-dimensional imaging of the mouse neurovasculature with magnetic resonance microscopy. PLoS One 6, e22643 (2011). 21818357 10.1371/journal.pone.0022643 PMC3144917

[b4] Xue S. *et al.* Indian-ink perfusion based method for reconstructing continuous vascular networks in whole mouse brain. PLoS One 9, e88067 (2014). 24498247 10.1371/journal.pone.0088067 PMC3907580

[b5] Fischer R. S., Wu Y., Kanchanawong P., Shroff H. & Waterman C. M. Microscopy in 3D: a biologist’s toolbox. Trends Cell Biol 21, 682–691 (2011). 10.1016/j.tcb.2011.09.008 PMC3478882 22047760

[b6] Tsai P. S. *et al.* Correlations of neuronal and microvascular densities in murine cortex revealed by direct counting and colocalization of nuclei and vessels. J Neurosci 29, 14553–14570 (2009). 10.1523/JNEUROSCI.3287-09.2009 PMC4972024 19923289

[b7] Zhang M. *et al.* Synchrotron radiation imaging is a powerful tool to image brain microvasculature. Med Phys 41, 031907 (2014). 24593725 10.1118/1.4865784

[b8] Chen R., Liu P., Xiao T. & Xu L. X. X-ray Imaging for Non-Destructive Microstructure Analysis at SSRF. Adv Mater 26, 7688–7691 (2014). 25319475 10.1002/adma.201402956

[b9] Westneat M. W., Socha J. J. & Lee W. K. Advances in biological structure, function, and physiology using synchrotron X-ray imaging*. Annu Rev Physiol 70, 119–142 (2008). 18271748 10.1146/annurev.physiol.70.113006.100434

[b10] Fratini M. *et al.* Simultaneous submicrometric 3D imaging of the micro-vascular network and the neuronal system in a mouse spinal cord. Sci Rep 5, 8514 (2015). 25686728 10.1038/srep08514 PMC4649670

[b11] Zhang M. Q. *et al.* Three-dimensional visualization of rat brain microvasculature following permanent focal ischaemia by synchrotron radiation. Br J Radiol 87, 20130670 (2014). 24702152 10.1259/bjr.20130670 PMC4075551

[b12] Lang S. *et al.* Three-dimensional quantification of capillary networks in healthy and cancerous tissues of two mice. Microvasc Res 84, 314–322 (2012). 22796313 10.1016/j.mvr.2012.07.002

[b13] Heinzer S. *et al.* Novel three-dimensional analysis tool for vascular trees indicates complete micro-networks, not single capillaries, as the angiogenic endpoint in mice overexpressing human VEGF(165) in the brain. Neuroimage 39, 1549–1558 (2008). 18077185 10.1016/j.neuroimage.2007.10.054

[b14] dos Santos Rolo T., Ershov A., van de Kamp T. & Baumbach T. *In vivo* X-ray cine-tomography for tracking morphological dynamics. Proc Natl Acad Sci USA 111, 3921–3926 (2014). 24594600 10.1073/pnas.1308650111 PMC3964127

[b15] Shirai M. *et al.* Synchrotron radiation imaging for advancing our understanding of cardiovascular function. Circ Res 112, 209–221 (2013). 23287456 10.1161/CIRCRESAHA.111.300096

[b16] Bravin A., Coan P. & Suortti P. X-ray phase-contrast imaging: from pre-clinical applications towards clinics. Phys Med Biol 58, R1–35 (2013). 23220766 10.1088/0031-9155/58/1/R1

[b17] Wilkins S. W., Gureyev T. E., Gao D., Pogany A. & Stevenson A. W. Phase-contrast imaging using polychromatic hard X-rays. Nature 384, 335–338 (1996).

[b18] Paganin D. & Nugent K. A. Noninterferometric phase imaging with partially coherent light. Phys Rev Lett 80, 2586–2589 (1998).

[b19] Hofmann R., Moosmann J. & Baumbach T. Criticality in single-distance phase retrieval. Opt Express 19, 25881–25890 (2011). 22274176 10.1364/OE.19.025881

[b20] Heinzer S. *et al.* Hierarchical microimaging for multiscale analysis of large vascular networks. Neuroimage 32, 626–636 (2006). 10.1016/j.neuroimage.2006.03.043 16697665

[b21] Huang S. *et al.* In-line phase-contrast and grating-based phase-contrast synchrotron imaging study of brain micrometastasis of breast cancer. Sci Rep 5, 9418 (2015). 25818989 10.1038/srep09418 PMC4377630

[b22] Lin H. *et al.* Grating-based phase-contrast imaging of tumor angiogenesis in lung metastases. PLoS One 10, e0121438 (2015). 25811626 10.1371/journal.pone.0121438 PMC4374967

[b23] Zysk A. M. *et al.* Nondestructive volumetric imaging of tissue microstructure with benchtop x-ray phase-contrast tomography and critical point drying. Biomed Opt Express 3, 1924–1932 (2012). 22876355 10.1364/BOE.3.001924 PMC3409710

[b24] Hu J., Cao Y., Wu T., Li D. & Lu H. High-resolution three-dimensional visualization of the rat spinal cord microvasculature by synchrotron radiation micro-CT. Med Phys 41, 101904 (2014). 25281956 10.1118/1.4894704

[b25] Stolz E. *et al.* Angioarchitectural changes in subacute cerebral venous thrombosis. A synchrotron-based micro- and nano-CT study. Neuroimage 54, 1881–1886 (2011). 20974267 10.1016/j.neuroimage.2010.10.056

[b26] Tagami M. *et al.* Ultrastructural characteristics of occluded perforating arteries in stroke-prone spontaneously hypertensive rats. Stroke 18, 733–740 (1987). 3603600 10.1161/01.str.18.4.733

[b27] Duvernoy H. M., Delon S. & Vannson J. L. Cortical blood vessels of the human brain. Brain Res Bull 7, 519–579 (1981). 7317796 10.1016/0361-9230(81)90007-1

