# Supplementary material for: Ultra-high-resolution 3D digitalized imaging of the cerebral angioarchitecture in rats using synchrotron radiation
Source: Sci Rep. 2015 Oct 7;5:14982. doi: 10.1038/srep14982 (PMC4595735; doi:10.1038/srep14982)
Supplement: Supplementary Video Legend [file srep14982-s1.doc]

**Manuscript #:** SREP-15-02205B

**Title:** Ultra-high-resolution 3D digitalized imaging of the cerebral angioarchitecture in rats using synchrotron radiation

**Authors List:** Meng-Qi Zhang, Luo Zhou, Qian-Fang Deng, Yuan-Yuan Xie, Ti-Qiao Xiao, Yu-Ze Cao, Ji-Wen Zhang, Xu-Meng Chen, Xian-Zhen Yin, Bo Xiao

**Supplementary Video Legends**

**Supplementary Video 1.** Synchrotron-based virtual micro-endoscopy to reveal the trajectory of targeted vessels in 3D
